# Histological Comparison of Collagenated Cancellous Equine Bone Blocks Used as Inlay or Onlay for Lateral Bone Augmentation in Rabbits

**DOI:** 10.3390/ma16206742

**Published:** 2023-10-18

**Authors:** Ryuichi Sakaguchi, Samuel Porfirio Xavier, Kenzo Morinaga, Daniele Botticelli, Erick Ricardo Silva, Yasushi Nakajima, Shunsuke Baba

**Affiliations:** 1 Department of Oral Implantology, Osaka Dental University, 8-1 Kuzuhahanazonocho, Hirakata 573-1121, Japan; donchan99@me.com (R.S.); morinaga-k@cc.osaka-dent.ac.jp (K.M.); y.nakajima@me.com (Y.N.); baba-s@cc.osaka-dent.ac.jp (S.B.); 2 Department of Oral and Maxillofacial Surgery and Periodontology, Faculty of Dentistry of Ribeirão Preto, University of São Paulo, Av. do Café, Subsetor Oeste, 11 (N-11), Ribeirao Preto 14040-904, SP, Brazil; spx@forp.usp.br (S.P.X.); erickricardo.rp@gmail.com (E.R.S.); 3 ARDEC Academy, 47923 Rimini, Italy

**Keywords:** animal study, bone healing, histology, lateral augmentation, bone transplantation, biomaterial, bone defect

## Abstract

Background: The conformation of the recipient site for an inlay graft presents an increased contact with the parent bone compared to an onlay graft. This might favor bone growth within the inlay compared to onlay grafts. Hence, the objective of this study was to compare the bone incorporation and remodeling processes of xenogeneic en bloc grafts placed using two bone grafting techniques, i.e., onlay vs. inlay. Methods: In this prospective, randomized, split-mouth study (test and control sides in the same animal), two bone grafting techniques were comparatively evaluated. The lateral aspect of the rabbit mandible was used as the recipient site, bilaterally. On one side of the mandible, the cortical bone was perforated with drills to allow a better bone formation from the bone wound and the marrow spaces. A xenogeneic bone block was fixed in the center of the prepared region, representing the onlay site. On the other side of the mandible, a 7 mm wide and 3 mm deep circumferential defect was prepared using trephines and drills. A xenogeneic bone block was fixed in the center of the defect, representing the inlay site. Two healing periods were applied in the study: 2 and 10 weeks, each represented by 10 rabbits (*n* = 10 for each period). Results: After 2 weeks of healing, the mean percentage of new bone was 10.4% and 23.3% at the onlay and inlay grafts, respectively (*p* = 0.022). After 10 weeks of healing, new bone increased to 13.2% at the onlay sites and 25.4% at the inlay sites (*p* = 0.080). In the 10-week period, the inlay grafts presented a homogeneous growth of new bone in all regions, while in the onlay grafts, low percentages of new bone were observed in the external regions. Conclusion: The percentage of new bone increased faster and was higher in the inlay grafts than in the onlay grafts. This outcome might be related to the self-contained conformation of the recipient site in the inlay group, which offered more sources for new bone formation compared to the one-wall conformation of the recipient sites in the onlay group. The osteoconductive properties of the biomaterial allowed the newly formed bone to reach the most peripheral regions in both groups. The osteoconductive properties of the biomaterial, together with the protection offered by the collagen membrane, allowed marginal closure of the defects by newly formed bone in the inlay group.

## 1. Introduction

To reduce the loss of alveolar bone volume that occurs after tooth extraction [1,2], which is even more extensive in cases of damage to the buccal bone crest, biomaterials might be used to fill the alveoli [3]. In cases of multiple tooth extractions, extensive periodontitis, or trauma, reconstruction of the lost bone volume may be required. When defects reach a critical size, which is an extension that does not allow complete spontaneous healing [4,5], reconstruction becomes a challenge for surgeons [6]. Block grafting has become a surgical tool widely used in the treatment of mandibular bone defects resulting from traumatic injuries, resorption, tooth extraction, and birth defects [7]. The objective of this procedure is to restore lost bone volume, and it has been frequently used in the field of implant dentistry as a way of enabling the prosthetic rehabilitation of osseointegrated implants [8,9,10]. Some types of grafts are recommended for reconstruction of atrophic alveolar ridges. Autogenous, xenogeneic, and allogeneic grafts are the most used grafts [11,12,13,14].

Autogenous grafts are considered the “gold standard” because of their osteogenic and osteoconductive properties, which stimulate the process of bone incorporation [15,16,17,18,19]. However, they have disadvantages, such as surgical morbidity and longer operative time [20,21,22,23,24]. Autogenous grafting procedures can be quite complex, and success depends on the size of the bone defect and availability of the donor site. This has led to the development of new biomaterials capable of restoring lost bone volume in terms of quantity and quality [25,26,27]. Studies have shown that block autogenous bone grafts may be more resistant to bone volume loss in the long term than particulate grafts [28]. However, other studies have shown that block grafts are prone to resorption during repair [29]. Thus, new materials and techniques have been developed to reduce the high rate of graft resorption and maintain bone volume [28,30].

As a viable alternative for replacing autogenous bone, the xenogeneic graft has been used in bone volume augmentation procedures [31,32,33,34,35,36], as it presents clinical advantages such as a decrease in surgical morbidity, greater technical ease, and the ability to conform and adapt the block following the anatomy of the recipient bed [31,37,38,39]. To install the graft in the recipient bed, some surgical techniques can be used, such as bone grafts in onlay blocks, osteogenic distraction, and particulate bone grafts associated with titanium meshes or collagen membranes [39]. In a clinical study [40], the technique of onlay grafting of autogenous blocks collected from the iliac crest for the restoration of lost maxillary bone volume was applied in 30 patients. During the period of bone incorporation, extensive reabsorption of the total graft volume was observed.

In experimental studies [41], the influence of perforations of the recipient beds on grafting procedures using onlay blocks harvested from the iliac crests of rabbits was evaluated. The blocks installed on perforated beds presented a smaller reabsorption during the repair time owing to greater blood perfusion from the bone matrix to the graft, which allowed a higher number of proteins associated with revascularization and osteogenesis.

Faced with the difficulty of establishing a grafting technique capable of providing adequate bone incorporation, less resorption during the repair period, and maintenance of bone volume in the long term, this work aimed to comparatively evaluate the process of incorporation, resorption, and remodeling of xenogeneic block bone grafts using two surgical techniques: onlay vs. inlay graft techniques. The conformation of the recipient site for an inlay graft presents an increased contact with the parent bone compared to the onlay graft. This might favor graft integration and bone formation within the inlay compared to onlay grafts. Hence, the objective of this study was to compare the bone incorporation and remodeling processes of xenogeneic en bloc grafts, installed using two bone grafting techniques, i.e., onlay vs. inlay.

## 2. Materials and Methods

### 2.1. Ethical Statements

This research project was approved by the Committee on Ethics in the Use of Animals of the Faculty of Dentistry of Ribeirão Preto, University of São Paulo, Brazil, on 25 September 2019, protocol #2019.1.619.58.2. The proposed experimental procedures were carried out in accordance with the legislation for animal experimentation in Brazil. The ARRIVE Checklist was used in this study.

### 2.2. Study Design

**Figure 1:**
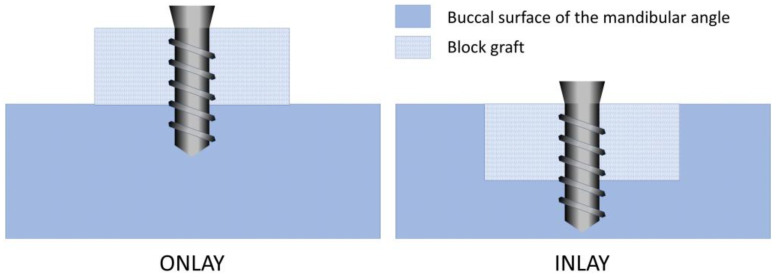
Schematic drawing showing the two procedures. For more information, see Supplementary Figure S1, which illustrates a histological image of the experimental region of the rabbit mandible.

In this prospective, randomized, split-mouth study (test and control sides in the same animal), two bone grafting techniques were comparatively evaluated on the lateral aspect of rabbit mandibles (Figure 1). One side was prepared with perforations (onlay site), whereas the other side was prepared with trephines and drills to obtain a standardized recipient site (inlay site). A xenogeneic bone block was fixed in the center with a titanium screw in both sides of the mandible and covered with a collagen membrane. Two healing periods were applied in the study: 2 and 10 weeks of healing.

### 2.3. Experimental Animals and Sample Size

Data from a study in which similar grafts were fixed to the bone plate of the lateral aspect of rabbit mandibles were used for sample calculation [42]. In this study, the grafts were either treated with argon plasma or left untreated. A maximum standard deviation of 7.4% was found. In the present study, a 10% difference between the onlay and inlay was considered clinically relevant. Consequently, with α = 0.05 and a power of 0.9, a sample size of eight pairs of animals was obtained to reject the null hypothesis that this response difference is zero (PS Power and Sample Size Calculations; by William D. Dupont and Walton D. Plummer). The sample size was increased to 10 for possible complications at various levels during the experiment. Hence, twenty adult male New Zealand White rabbits, 10 for each period of healing, weighing 3.5–4.0 kg and aged between 5 and 6 months, were included in the study.

### 2.4. Randomization and Allocation Concealment

Randomization was performed electronically by an author who was not involved in the selection of animals or any surgical procedure (S.P.X.). The treatment allocation was maintained in a sealed opaque envelope that was opened immediately before the fixation of the first graft. The histological examinator was not informed of the time of healing and treatment allocation, and the histological slides were coded. However, the conformation of the sites could be recognized on the histological slides.

### 2.5. Biomaterials

SpBlock is a rigid dried block composed of collagenated cancellous equine bone (Tecnoss, Giaveno, Italy). It is produced using an exclusive process that avoids the ceramization of hydroxyapatite crystals, thus accelerating physiological resorption.

Bio-Guide (Geistlich Biomaterials, Wolhusen, LU, Switzerland) is a porcine-derived resorbable membrane composed of types I and III collagen. It is composed of a bilayer structure with a smooth outer layer aimed at avoiding the progression of soft tissues within the region to be regenerated, and a porous inner layer that aims to favor bone cells and vessel growth [43].

### 2.6. Anesthetic Procedures

The surgical procedures were performed under general anesthesia with intramuscular acepromazine (1.0 mg/kg; Acepran, Vetnil, Louveira, São Paulo, Brazil) and xylazine (3.0 mg/kg; Laboratórios Calier S/A, Barcelona, Spain) combined with ketamine I.M. (50.0 mg/kg; União Química Farmacêutica Nacional S/A, Embuguaçú, São Paulo, Brazil) 15 min after acepromazine administration. When properly sedated, animals were subjected to prophylactic antibiotic therapy with oxytetracycline (0.2 mL/kg; Biovet; Vargem Grande Paulista, São Paulo, Brazil). The area to be operated was shaved, and antisepsis was performed by topical application of 1% polyvinylpyrrolidone iodine solution (Riodeíne Tintura, Rioquímica, São José do Rio Preto, São Paulo, Brazil). Local anesthesia was performed with 2% mepivacaine and 1:100,000 noradrenaline (Mepinor, Nova DFL, Rio de Janeiro, Brazil).

### 2.7. Surgical Procedure

All surgeries were performed by a single operator (V.F.B.; see acknowledgments) who was experienced and qualified for the task.

**Figure 2:**
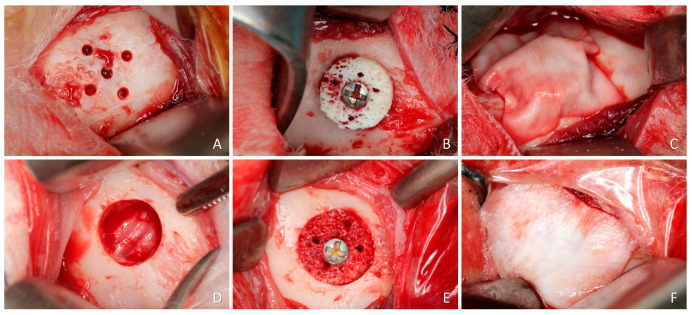
(**A**–**C**) Clinical photographs of the onlay procedure. (**A**) Perforations of the cortical layer. (**B**) Block graft secured with a screw. Note the perforations of the block. (**C**) Collagen membrane placed on the top of the block. (**D**–**F**) Clinical photographs of the inlay procedure. (**D**) Preparation of a calibrated defect, 7 mm wide and 3 mm deep. (**E**) Block graft inserted into the defect and secured with a screw. Note the perforations of the block. (**F**) Collagen membrane placed on the top of the block.

A linear incision of 2.5 to 3 cm was made bilaterally on the skin at the lower border of the mandible. Muscles and periosteum were reflected, and the flap was raised, exposing the convex bone of the buccal surface of the mandibular angle. At one site, a 1.0 mm truncated-conical drill coupled in a straight piece was used to perform five equidistant monocortical perforations in the entire diameter of the graft guided by a template made of stainless steel. These perforations reached the medullary portion of the recipient bed to promote blood and cell supply to the graft from the endosteum (Figure 2A). The xenogeneic bone block, 7 mm in diameter and 3 mm in height, was appositionally fixed (onlay; Figure 2B) using a 1.5 × 10 mm titanium screw (Neodent, Curitiba, Brazil).

On the other side of the mandible, a bone defect 7 mm wide and 3 mm deep was prepared using a trephine and refined with a diamond drill. (Figure 2D). Subsequently, the xenogeneic bone block was positioned inside the defect, adapted to the level of the adjacent bone margin, and secured with a titanium screw (Figure 2E). Collagen membranes (BioGuide^®^; Geistlich, Wolhusen, Switzerland) were used to cover both blocks (Figure 2C,F). Wound closure was performed with Vicryl 4-0 on the muscular planes, and Nylon 4-0 on the skin with simple stitches.

### 2.8. Animal Maintenance

All animals were medicated with ketoprofen (3.0 mg/kg, 12/12 h, i.m., 10% Ketofen; Merial, Campinas, São Paulo, Brazil) and 2% tramadol hydrochloride (1.0 mg/kg, 12/12 h, subcutaneous; Cronidor, Agener União Saúde Animal, Apucarana, Paraná, Brazil) in the postoperative period and in the following 3 days.

Animals were housed in the Animal Facility of the Faculty of Dentistry of Ribeirão Preto, University of São Paulo. They were kept in individual metal cages (1 animal/4500 cm²) in an acclimatized room with split air conditioning, an exhaust fan (27 to 34 air changes/h), and automatic lighting control (12 h light-dark cycle). All animals were fed with dedicated food and had access to water ad libitum.

A rigorous protocol for monitoring the animals was carried out throughout the experimental period, paying daily attention to the basic biological functions, feeding and excretion, behavioral signs in relation to postoperative pain, and monitoring of post-surgical infections and surgical wounds for suture care, bleeding, and/or signs of infection.

### 2.9. Euthanasia

The animals were euthanized by administering an overdose (2.0 mL) of intravenous thiopental 1.0 g (Thiopentax; Cristália, Itapira, São Paulo, Brazil) after 2 or 10 weeks, with 10 animals in each group. The experimental regions were dissected and reduced to individual blocks and maintained in 10% paraformaldehyde for fixation.

### 2.10. Histological Processing

The specimens were taken to the FORP-USP hard tissue section laboratory for histological preparation. Initially, the specimens were washed with running water to completely remove the fixing agent, dehydrated in a gradual and increasing sequence of ethyl alcohol, changed every three days under constant agitation (60%, 80%, 96%, and absolute alcohol twice), and subsequently embedded in resin (LR WhiteTM HardGrid; London Resin Co Ltd., Berkshire, UK) for impregnation and subsequent polymerization in an oven at 60 °C.

Once polymerization was complete, each block was cut following a transaxial plane in the center of the block guided by the fixation screw positioned at the center of the graft at the time of surgery.

Two sections of approximately 100–150 µm thickness were prepared using precision cutting/grinding equipment (Exakt; Apparatebau, Norderstedt, Germany) and ground to a thickness of approximately 60–80 µm. Histological sections were stained with either Toluidine Blue or with Stevenel’s Blue and Alizarin Red.

### 2.11. Histomorphometric Evaluation

For histological and histomorphometric evaluation, an expert evaluator (K.A.A.A., see acknowledgements) who did not participate in the other stages of the study, was calibrated with another expert (D.B.) until the inter-rater agreement achieved a Cohen’s coefficient of k > 0.90.

**Figure 3:**
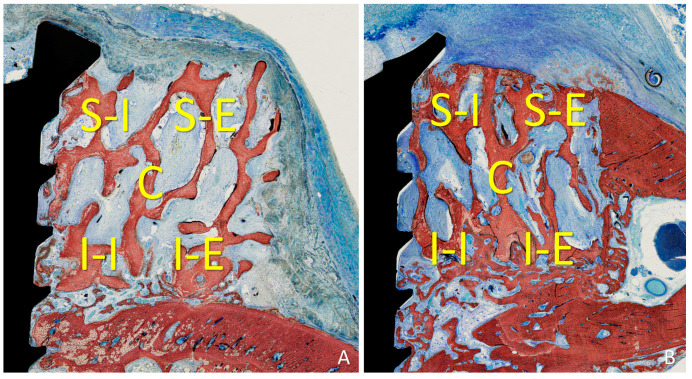
Five locations were evaluated within the grafted region lateral to the fixation screw: inferior/internal (I-I), inferior/external (I-E), superior/internal (S-I), superior/external (S-E), and central (C). (**A**) onlay graft; (**B**) inlay graft.

Five locations were evaluated within the grafted region lateral to the fixation screw: inferior/internal (I-I), inferior/external (I-E), superior/internal (S-I), superior/external (S-E), and central (C). The following tissues were assessed: new bone, xenograft, soft tissues (marrow spaces, provisional matrix, dense and loose tissues, and connective tissue), vessels, inflammatory infiltrate, and osteoclastic zones (Figure 3A,B). One grid containing 16 × 12 squares with dimensions of 75 µm (1200 × 900 microns; 1.08 mm^2^) was superimposed onto the photomicrographs of each region using NIS-Elements software (v. 5.11.01, Nikon, Tokyo, Japan).

### 2.12. Experimental Outcomes and Statistical Methods

The values obtained are expressed as mean ± standard deviation. The primary variable was the mineralized new bone. The secondary variables were other tissues evaluated in the morphometric analysis. The Shapiro–Wilk test was used to determine the normality of data and, according to the results, the differences between the test and control sites were evaluated by a paired *t*-test or a Wilcoxon matched-pairs signed rank test. GraphPad Prism (version 10.0.2 for Windows, GraphPad Software, Boston, MA, USA) was used for the statistical analysis. The significance level was 5%.

## 3. Results

### 3.1. Clinical Outcomes

The healing of the animals was uneventful. All histological slides were available for analysis, with *n* = 10 for both periods.

### 3.2. Descriptive Histological Evaluation

**Figure 4:**
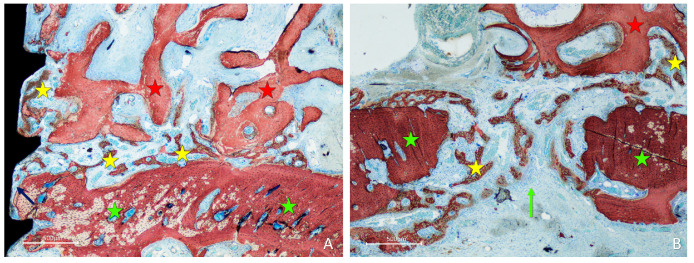
Photomicrographs of ground sections showing healing aspects at onlay graft sites after 2 weeks. (**A**) New bone formed from the recipient site and from the perforation of the fixation screw. (**B**) Active osteogenesis through cortical perforations. Landmarks indicating examples of tissues: yellow stars, new bone; red stars, graft; green stars, recipient site; green arrow, perforation of the recipient site; blue arrow, perforation of the fixation screw. Stevenel’s blue and alizarin red were used as stains.

After two weeks of healing, in the onlay group, new bone was found formed from the cortical layer of the lateral aspect of the mandible, interposed between the graft and parent bone, penetrating the cavities of the graft and lining onto the trabeculae (Figure 4A). Active osteogenesis was observed through the cortical perforations, which contributed to bone growth (Figure 4B).

**Figure 5:**
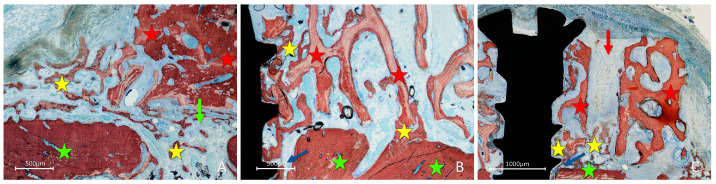
Photomicrographs of ground sections showing healing aspects at onlay graft sites after 2 weeks. New bone formed: (**A**) laterally to the graft and from the perforations of the cortical layer; (**B**) from the fixation screw site; (**C**) inside a perforation created in the graft. Landmarks indicating examples of tissues: yellow stars, new bone; red stars, graft; green stars, recipient site; green arrow, perforation of the recipient site; blue arrows, perforation of the fixation screw; red arrow, perforation of the graft. Stevenel’s blue and alizarin red were used as stains.

New bone was also observed forming laterally to the graft, progressing onto the outer surface of the graft (Figure 5A). New bone was also noted at the perforation of the fixation screw (Figure 5B) and inside the perforations created in the grafts (Figure 5C).

**Figure 6:**
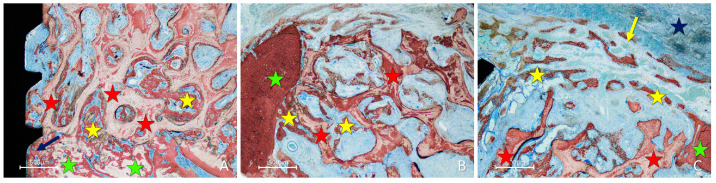
Photomicrographs of ground sections showing healing aspects at inlay graft sites after 2 weeks. Bone formation was observed: (**A**) from the cortex of the mandible and the perforations of the fixation screw; (**B**) from the lateral walls of the defect; (**C**) from the lateral walls of the defect growing over the top of the graft and underneath the collagen membrane. Landmarks indicating examples of tissues: yellow stars, new bone; red stars, graft; green stars ((**A**), base and (**B**,**C**), lateral wall of the defect); blue star, collagen membrane residues (**C**); blue arrow, perforation of the fixation screw; yellow arrow, new bone at the top of the defect. Stevenel’s blue and alizarin red were used as stains.

In the inlay group, after 2 weeks of healing, the position within the self-contained defect allowed the presence of multiple sources of new bone. Bone formation was observed in the cortex of the mandible, the perforations of the fixation screw (Figure 6A), and at the lateral aspects of the defect bone walls (Figure 6B). At the periphery of the defect, new bone formed from the cortical layer grew over the top of the graft and underneath the collagen membrane (Figure 6B,C), closing the entrance of the defect.

**Figure 7:**
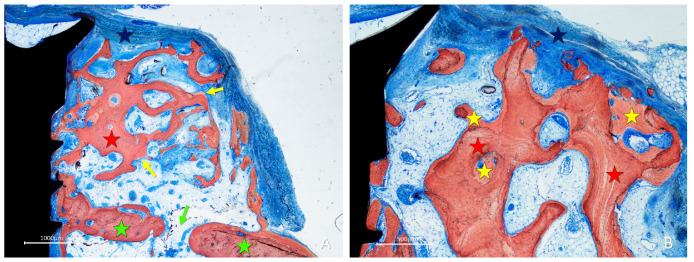
Photomicrographs of ground sections showing healing aspects at onlay graft sites after 10 weeks. (**A**) The space included among the trabeculae was filled with bone marrow and provisional matrix. (**B**) Note the new bone deposed onto the trabeculae. Landmarks indicating examples of tissues: yellow stars, new bone; red stars, graft; green stars, recipient site; blue stars, membrane; yellow arrows, newly formed bone laid on the graft trabeculae; green arrow, perforation of the fixation screw. Stevenel’s blue and alizarin red were used as stains.

After 10 weeks of healing, in the onlay group, bone was found lining the surface of the trabeculae (Figure 7A,B), while the spaces included among the trabeculae were filled with bone marrow and provisional matrix.

**Figure 8:**
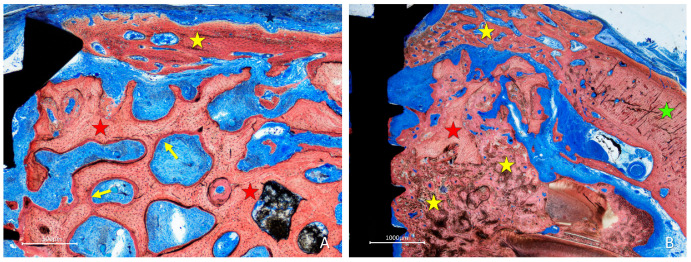
Photomicrographs of ground sections showing healing aspects at inlay graft sites after 10 weeks. (**A**) In some instances, a dense provisional matrix tissue was occupying large regions within the trabeculae of the graft that appeared to be lined by newly formed bone. (**B**) In other instances, a dense bone was included in the graft region, embedded to the graft trabeculae. (**A**,**B**) In several cases, the graft was covered by newly formed bone. Landmarks indicating examples of tissues: yellow stars, new bone; red stars, graft; green star, lateral wall of the recipient site; yellow arrows, newly formed bone laid on the graft trabeculae. Stevenel’s blue and alizarin red were used as stains.

In the inlay group, in most cases, the bone lined the trabeculae of the graft while the intra-trabecular spaces were filled with a dense provisional matrix or bone marrow (Figure 8A). In some instances, dense bone occupied a large part of the graft (Figure 8B); in several cases, the graft was covered by newly formed bone which was closing the entrance of the defect (Figure 8A,B).

### 3.3. Histomorphometric Assessments

**Figure 9:**
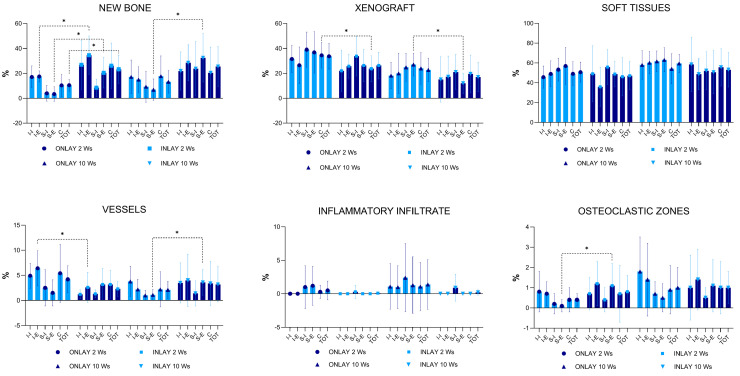
Tissue percentages in the various regions examined after 2 and 10 weeks (Ws) of healing. *, *p* < 0.05. (Graphs produced with GraphPad Prism).

After two weeks of healing, higher amounts of new bone were found in the inlay than in the onlay grafts (Figure 9). The differences were statistically significant for the mean total, I-E, and S-E regions. The regions with the lowest amount of new bone were S-I and S-E in the onlay graft and S-I in the inlay graft, i.e., the regions farthest from the bone walls. The xenograft was found at a lower percentage in the inlay grafts than in the onlay grafts, and the difference was statistically significant only for the C region.

The soft tissue percentage was similar in both groups, whereas that of the vessels was slightly higher in the onlay than in the inlay grafts in all regions. The difference was only statistically significant in the I-E region. Small amounts of inflammatory infiltrates and osteoclast zones were also observed.

After 10 weeks of healing, the mean total new bone of both groups increased slightly compared with the previous period examined (Figure 9). While the inferior regions, closer to the bone walls, mostly presented a tendency to decrease the proportion of new bone, in the peripheral regions (superior), new bone increased considerably in percentage for both grafts. The difference was statistically significant only in the S-E region. The percentage of xenograft decreased between the two evaluation periods, while the number of vessels decreased in the onlay graft group but increased in the inlay graft group. The percentage of inflammatory infiltrate increased only in the inlay group, while osteoclastic activity slightly increased in both grafts.

## 4. Discussion

The results from the present experiment showed that the mean total percentage of new bone was about double in the inlay compared to the onlay grafts in both periods of healing. However, the difference was statistically significant only after two weeks (*p* = 0.022), and not after 10 weeks of healing (*p* = 0.080). This lack of significance may be related to the high variability observed among the animals (Figure 9). However, statistically significant differences were observed (*p* = 0.027) when the data for the superior regions (SI + SE) were merged. In the various regions evaluated within both grafts, the proportion of new bone was always higher in the inlay grafts than in the onlay grafts. This difference is related to the placement of the inlay within a self-contained defect that presents multiple sites for bone formation. The defect was 7 mm wide, and its dimensions significantly affected healing.

In a study in rabbits, full-thickness bone defects 11 mm in diameter were created in the mandible [4]. The defects were either filled with autogenous bone or biphasic calcium phosphate granules or left empty. The evaluations performed after 4 and 12 weeks revealed incomplete healing in all empty specimens. Conversely, both the autogenous bone and biphasic calcium phosphate groups showed better results, with the percentage of new bone being superior in the former than in the latter. It should be emphasized that in that study, through–through defects were applied such that new bone could only be formed from the edges of the defect. The defects used in the present study were slightly smaller and did not involve the full thickness of the mandibular bone, hence presenting a base of bone that also contributed to bone formation. This, in turn, means that healing was more favorable in the present model than that described in the study cited above, providing more bone walls from which bone is generated. This also resulted in more rapid formation of new bone within the inlay graft than in the onlay graft. In fact, in the onlay graft, bone could only be formed from the base of the defect, whereas in the inlay graft, bone could be formed from the base and lateral walls of the defect.

The tendency of circumferential defects to heal spontaneously with [44] or without implants [45] might have also influenced the results. Indeed, 7.3 mm wide circumferential peri-implant marginal defects have been proven to heal without filler material in an experiment on the alveolar bone ridge of dogs [46]. Nevertheless, the osteoconductive properties of the biomaterial used represent a key factor in achieving proper healing.

In an experiment in dogs, a block graft composed of deproteinized bovine bone mineral (DBBM) and a block of autogenous bone were fixed to the lateral aspect of the mandible [47]. After six months of healing, the autogenous block was found to be incorporated into the lateral aspect of the mandible, while the DBBM block presented little newly formed bone at the base of the graft, close to the recipient site. In another experiment [48,49], standardized defects were created in the lateral aspect of the dog mandibles. After 3 months, DBBM or autogenous blocks were placed and secured with fixation screws within the bone defects. Three months later, at all sites, one implant was placed in the region of the interface between the graft and recipient sites. The results after three months showed that all implants were integrated. However, the buccal aspects of the implant, that is, the aspect in contact with the graft, only presented integration in the autogenous group, whereas almost no integration was observed in the DBBM graft [48]. Moreover, graft integration has been evaluated [49]. While a vital autogenous block was well-integrated into the parent bone, the DBBM block was mainly separated from the parent bone by a layer of connective tissue. In only a few instances, the bone occupied the graft in the basal zones.

In the present study, collagenated cancellous equine bone was used. New bone growing within the graft, lining the trabeculae at the basal aspect of the graft, was found after 2 weeks of healing. After 10 weeks, new bone was formed within the entire body of the graft toward the surface. This outcome agrees with that reported in another experiment in which similar equine graft blocks, either treated with plasma argon or left untreated, were fixed onto the lateral side of the mandible of rabbits [42]. Similar to the present study, in that experiment, the bone grew within the graft onto the trabeculae, up to the surface. In another rabbit experiment [34], equine block grafts were compared with autogenous block grafts. While the two showed similar percentages of newly formed bone, volume maintenance and graft incorporation were better in the autologous compared to the xenogeneic graft.

Analyses performed in different regions of the grafts allowed for further speculation. In both periods of healing, new bone was formed at high percentages in all but the superior regions of the inlay grafts. In practice, when lateral augmentation is performed on an alveolar bone with a flat surface (without a self-contained defect), the most lateral region of the graft might present very little bone, influencing the integration of implants in this zone.

A systematic review evaluated horizontal alveolar ridge augmentation by using allogeneic bone blocks. Similar outcomes have been observed in implant treatment between allogeneic and autogenous bone blocks [50,51]. Systematic review showed that tooth blocks might also represent an alternative to autogenous bone block grafts [52,53].

In the present study, new bone formation was observed between the base of the graft and recipient bone site. Owing to the convexity of the mandible in the region of interest, the adaptation of the graft onto the surface was not completely congruent, resulting in gaps. Nevertheless, these gaps were filled with newly formed bone, as has already been described in another study mentioned above [42]. The healing in the interface between the gap and recipient site has been thoroughly described in a study that evaluated the healing of autogenous bone block grafts collected from the calvaria and secured either with a “position” or a “lag” screw technique [54]. It was shown that, in this specific region, new bone that was present in a low percentage after 2 weeks increased progressively such that, after 40 days, the graft was completely incorporated into the parent bone of the mandible.

In the present study, perforations produced at the recipient sites showed active osteogenesis. This activity has been described in detail in a previous study [52]. The influence of perforations at the recipient sites was evaluated in an experimental study on 36 rabbits [41]. Autogenous blocks harvested from the iliac crest were grafted to the lateral aspect of the mandible. One side was drilled while the control site was left intact. The healing was studied after 3, 5, 7, 10, 20, and 60 days. The authors concluded that perforations induced angiogenesis earlier than in the control sites, allowing for greater remodeling and higher bone graft density.

In the present study, the biomaterial was reduced by approximately 1/3 between weeks 2 and 10. However, it was still present at a high percentage after 10 weeks in both the grafts. The slightly increased number of osteoclasts in the 10- compared to the 2-week healing period substantiates the speculation that graft resorption was still in progress. These results are similar to those reported in a similar study [42]. In that study, the percentage of xenograft decreased by approximately 1/3 between weeks 2 and 10. However, in that study, the peak of osteoclast presence was seen after 6 weeks.

The percentage of soft tissues increased between weeks 2 and 10. This was partly due to the remodeling of the newly formed bone and the formation of immature and mature bone marrow, as well as the invasion of the external regions of the graft by connective tissue.

Several limitations should be mentioned regarding the present study, such as the model used, the position of the graft, which is quite different from the alveolar bone, and the faster healing speed of rabbits compared to humans. Longer healing periods should be also analyzed. Based on the results of the present study, clinical studies should be performed to evaluate healing in humans.

## 5. Conclusions

The percentage of new bone increased faster and was higher in the inlay grafts than in the onlay grafts. This outcome might be related to the self-contained conformation of the recipient site in the inlay group, which offered more sources for new bone formation compared to the one-wall conformation of the recipient sites in the onlay group. The osteoconductive properties of the biomaterial allowed the newly formed bone to reach the most peripheral regions in both the groups. The osteoconductive properties of the biomaterial, together with the protection offered by the collagen membrane, allowed marginal closure of the defects by newly formed bone in the inlay group.

## Data Availability

The data are available following a reasonable request.

## Supplementary Materials

The following supporting information can be downloaded at: https://www.mdpi.com/article/10.3390/ma16206742/s1, Figure S1: Photomicrograph of a ground section illustrating a section of a rabbit mandible with the various bone compartments. The dotted regions indicate the position of onlay/inlay grafts.

## References

[1] Araújo M.G., Lindhe J. (2005). Dimensional ridge alterations following tooth extraction. An experimental study in the dog. J. Clin. Periodontol..

[2] Schropp L., Wenzel A., Kostopoulos L., Karring T. (2003). Bone healing and soft tissue contour changes following single-tooth extraction: A clinical and radiographic 12-month prospective study. Int. J. Periodontics Restor. Dent..

[3] Avila-Ortiz G., Elangovan S., Kramer K.W., Blanchette D., Dawson D.V. (2014). Effect of alveolar ridge preservation after tooth extraction: A systematic review and meta-analysis. J. Dent. Res..

[4] Kotagudda Ranganath S., Schlund M., Delattre J., Ferri J., Chai F. (2022). Bilateral double site (calvarial and mandibular) critical-size bone defect model in rabbits for evaluation of a craniofacial tissue engineering constructs. Mater. Today Biol..

[5] Schmitz J.P., Hollinger J.O. (1986). The critical size defect as an experimental model for craniomandibulofacial nonunions. Clin. Orthop. Relat. Res..

[6] Marx R.E. (2007). Bone and bone graft healing. Oral Maxillofac. Surg. Clin. North. Am..

[7] Habal M.B. (1994). Bone grafting in craniofacial surgery. Clin. Plast. Surg..

[8] Adell R., Lekholm U., Rockler B., Brånemark P.I. (1981). A 15-year study of osseointegrated implants in the treatment of the edentulous jaw. Int. J. Oral Surg..

[9] Albrektsson T., Dahl E., Enbom L., Engevall S., Engquist B., Eriksson A.R., Feldmann G., Freiberg N., Glantz P.O., Kjellman O. (1988). Osseointegrated oral implants. A Swedish multicenter study of 8139 consecutively inserted Nobelpharma implants. J. Periodontol..

[10] Jemt T., Lekholm U., Gröndahl K. (1990). 3-year followup study of early single implant restorations ad modum Brånemark. Int. J. Periodontics Restor. Dent..

[11] Misch C.M., Misch C.E., Resnik R.R., Ismail Y.H. (1992). Reconstruction of maxillary alveolar defects with mandibular symphysis grafts for dental implants: A preliminary procedural report. Int. J. Oral Maxillofac. Implant..

[12] Misch C.E. (1990). Divisions of available bone in implant dentistry. Int. J. Oral Implantol..

[13] Salvato G., Agliardi E. (2007). Calvarial bone grafts in severe maxillary atrophy: Preprosthetic surgery with sedation. Implant. Dent..

[14] Le B., Burstein J., Sedghizadeh P.P. (2008). Cortical tenting grafting technique in the severely atrophic alveolar ridge for implant site preparation. Implant. Dent..

[15] Donovan M.G., Dickerson N.C., Mitchell J.C. (1994). Calvarial bone harvest and grafting techniques for maxillary and mandibular implant surgery. Atlas Oral Maxillofac. Surg. Clin. N. Am..

[16] von Arx T., Cochran D.L., Hermann J.S., Schenk R.K., Higginbottom F.L., Buser D. (2001). Lateral ridge augmentation and implant placement: An experimental study evaluating implant osseointegration in different augmentation materials in the canine mandible. Int. J. Oral Maxillofac. Implant..

[17] von Arx T., Cochran D.L., Hermann J.S., Schenk R.K., Buser D. (2001). Lateral ridge augmentation using different bone fillers and barrier membrane application. A histologic and histomorphometric pilot study in the canine mandible. Clin. Oral Implant. Res..

[18] Chiriac G., Herten M., Schwarz F., Rothamel D., Becker J. (2005). Autogenous bone chips: Influence of a new piezoelectric device (Piezosurgery) on chip morphology, cell viability and differentiation. J. Clin. Periodontol..

[19] Nowzari H., Aalam A.A. (2007). Mandibular cortical bone graft part 2: Surgical technique, applications, and morbidity. Compend. Contin. Educ. Dent..

[20] Chiapasco M., Zaniboni M., Boisco M. (2006). Augmentation procedures for the rehabilitation of deficient edentulous ridges with oral implants. Clin. Oral Implant. Res..

[21] Nkenke E., Weisbach V., Winckler E., Kessler P., Schultze-Mosgau S., Wiltfang J., Neukam F.W. (2004). Morbidity of harvesting of bone grafts from the iliac crest for preprosthetic augmentation procedures: A prospective study. Int. J. Oral Maxillofac. Surg..

[22] Nkenke E., Schultze-Mosgau S., Radespiel-Tröger M., Kloss F., Neukam F.W. (2001). Morbidity of harvesting of chin grafts: A prospective study. Clin. Oral Implant. Res..

[23] von Arx T., Häfliger J., Chappuis V. (2005). Neurosensory disturbances following bone harvesting in the symphysis: A prospective clinical study. Clin. Oral Implant. Res..

[24] McAllister B.S., Haghighat K. (2007). Bone augmentation techniques. J. Periodontol..

[25] Buser D., Chappuis V., Belser U.C., Chen S. (2017). Implant placement post extraction in esthetic single tooth sites: When immediate, when early, when late?. Periodontology 2000.

[26] Sheikh Z., Najeeb S., Khurshid Z., Verma V., Rashid H., Glogauer M. (2015). Biodegradable Materials for Bone Repair and Tissue Engineering Applications. Materials.

[27] Cicciù M., Cervino G., Herford A.S., Famà F., Bramanti E., Fiorillo L., Lauritano F., Sambataro S., Troiano G., Laino L. (2018). Facial Bone Reconstruction Using both Marine or Non-Marine Bone Substitutes: Evaluation of Current Outcomes in a Systematic Literature Review. Mar. Drugs.

[28] Dasmah A., Thor A., Ekestubbe A., Sennerby L., Rasmusson L. (2012). Particulate vs. block bone grafts: Three-dimensional changes in graft volume after reconstruction of the atrophic maxilla, a 2-year radiographic follow-up. J. Craniomaxillofac. Surg..

[29] Nyström E., Ahlqvist J., Legrell P.E., Kahnberg K.E. (2002). Bone graft remodelling and implant success rate in the treatment of the severely resorbed maxilla: A 5-year longitudinal study. Int. J. Oral Maxillofac. Surg..

[30] Gordh M., Alberius P. (1999). Some basic factors essential to autogeneic nonvascularized onlay bone grafting to the craniofacial skeleton. Scand. J. Plast. Reconstr. Surg. Hand Surg..

[31] Pistilli R., Felice P., Piatelli M., Nisii A., Barausse C., Esposito M. (2014). Blocks of autogenous bone versus xenografts for the rehabilitation of atrophic jaws with dental implants: Preliminary data from a pilot randomised controlled trial. Eur. J. Oral Implantol..

[32] Al Ruhaimi K.A. (2001). Bone graft substitutes: A comparative qualitative histologic review of current osteoconductive grafting materials. Int. J. Oral Maxillofac. Implant..

[33] Troeltzsch M., Troeltzsch M., Kauffmann P., Gruber R., Brockmeyer P., Moser N., Rau A., Schliephake H. (2016). Clinical efficacy of grafting materials in alveolar ridge augmentation: A systematic review. J. Craniomaxillofac. Surg..

[34] Silva E.R., Balan V.F., Botticelli D., Soldini C., Okamoto R., Xavier S.P. (2021). Histomorphometric, Immunohistochemical and Microtomographic Comparison between Autogenous and Xenogenous Bone Blocks for Mandibular Lateral Augmentation in Rabbits. Materials.

[35] Romito G.A., Villar C.C., Sapata V.M., Soares H.H., Fonseca M.A., Conde M., Hammerle C.H.F., Schwarz F. (2022). Autogenous bone block versus collagenated xenogeneic bone block in the reconstruction of the atrophic alveolar ridge: A non-inferiority randomized clinical trial. J. Clin. Periodontol..

[36] Romito G.A., Soares H.H., do Amaral G.C.L.S., Fonseca M.A., Sapata V.M., Conde M.C., Hammerle C.H.F., Schwarz F., Villar C.C. (2023). Radiographic outcomes of ridge reconstruction with autogenous bone block versus collagenated xenogeneic bone block: A randomized clinical trial. Clin. Oral Implant. Res..

[37] Kolk A., Handschel J., Drescher W., Rothamel D., Kloss F., Blessmann M., Heiland M., Wolff K.D., Smeets R. (2012). Current trends and future perspectives of bone substitute materials—From space holders to innovative biomaterials. J. Craniomaxillofac. Surg..

[38] Esposito M., Grusovin M.G., Felice P., Karatzopoulos G., Worthington H.V., Coulthard P. (2009). The efficacy of horizontal and vertical bone augmentation procedures for dental implants—A Cochrane systematic review. Eur. J. Oral Implantol..

[39] Xuan F., Lee C.U., Son J.S., Fang Y., Jeong S.M., Choi B.H. (2014). Vertical ridge augmentation using xenogenous bone blocks: A comparison between the flap and tunneling procedures. J. Oral Maxillofac. Surg..

[40] Nyström E., Legrell P.E., Forssell A., Kahnberg K.E. (1995). Combined use of bone grafts and implants in the severely resorbed maxilla. Postoperative evaluation by computed tomography. Int. J. Oral Maxillofac. Surg..

[41] Faria P.E., Okamoto R., Bonilha-Neto R.M., Xavier S.P., Santos A.C., Salata L.A. (2008). Immunohistochemical, tomographic and histological study on onlay iliac grafts remodeling. Clin. Oral Implant. Res..

[42] Kanayama M., Botticelli D., Apaza Alccayhuaman K.A., Yonezawa D., Silva E.R., Xavier S.P. (2021). The Impact on the Healing of Bioactivation with Argon Plasma of a Xenogeneic Graft with Adequate Fixation but Poor Adaptation to the Recipient Site: An Experimental Study in Rabbits. Int. J. Oral Maxillofac. Implant..

[43] Schwarz F., Sager M., Ferrari D., Mihatovic I., Becker J. (2009). Influence of recombinant human platelet-derived growth factor on lateral ridge augmentation using biphasic calcium phosphate and guided bone regeneration: A histomorphometric study in dogs. J. Periodontol..

[44] Botticelli D., Berglundh T., Buser D., Lindhe J. (2003). The jumping distance revisited: An experimental study in the dog. Clin. Oral Implant. Res..

[45] Carmagnola D., Berglundh T., Lindhe J. (2002). The effect of a fibrin glue on the integration of Bio-Oss with bone tissue. A experimental study in labrador dogs. J. Clin. Periodontol..

[46] Botticelli D., Berglundh T., Lindhe J. (2004). Resolution of bone defects of varying dimension and configuration in the marginal portion of the peri-implant bone. An experimental study in the dog. J. Clin. Periodontol..

[47] Araújo M.G., Sonohara M., Hayacibara R., Cardaropoli G., Lindhe J. (2002). Lateral ridge augmentation by the use of grafts comprised of autologous bone or a biomaterial: An experiment in the dog. J. Clin. Periodontol..

[48] De Santis E., Lang N.P., Scala A., Viganò P., Salata L.A., Botticelli D. (2012). Healing outcomes at implants installed in grafted sites: An experimental study in dogs. Clin. Oral Implant. Res..

[49] De Santis E., Lang N.P., Favero G., Beolchini M., Morelli F., Botticelli D. (2015). Healing at mandibular block-grafted sites: An experimental study in dogs. Clin. Oral Implant. Res..

[50] Starch-Jensen T., Deluiz D., Tinoco E.M.B. (2020). Horizontal Alveolar Ridge Augmentation with Allogeneic Bone Block Graft Compared with Autogenous Bone Block Graft: A Systematic Review. J. Oral Maxillofac. Res..

[51] Donkiewicz P., Benz K., Kloss-Brandstätter A., Jackowski J. (2021). Survival Rates of Dental Implants in Autogenous and Allogeneic Bone Blocks: A Systematic Review. Medicina.

[52] Starch-Jensen T., Vitenson J., Deluiz D., Østergaard K.B., Tinoco E.M.B. (2022). Lateral Alveolar Ridge Augmentation with Autogenous Tooth Block Graft Compared with Autogenous Bone Block Graft: A Systematic Review. J. Oral Maxillofac. Res..

[53] Guan D., Zhao R., Guo Y., Li J., Ma N., Gong J. (2023). Efficacy of autogenous tooth block for lateral ridge augmentation compared with autogenous bone block: A systematic review and meta-analysis. Medicine.

[54] Caneva M., Botticelli D., Carneiro Martins E.N., Caneva M., Lang N.P., Xavier S.P. (2017). Healing at the interface between recipient sites and autologous block bone grafts affixed by either position or lag screw methods: A histomorphometric study in rabbits. Clin. Oral Implant. Res..

