# Supplementary material for: Histological Comparison of Collagenated Cancellous Equine Bone Blocks Used as Inlay or Onlay for Lateral Bone Augmentation in Rabbits
Source: Materials (Basel). 2023 Oct 18;16(20):6742. doi: 10.3390/ma16206742 (PMC10608602; doi:10.3390/ma16206742)
Supplement: Supplementary file 1 [file materials-16-06742-s001.zip › materials-2629876-supplementary.pdf]

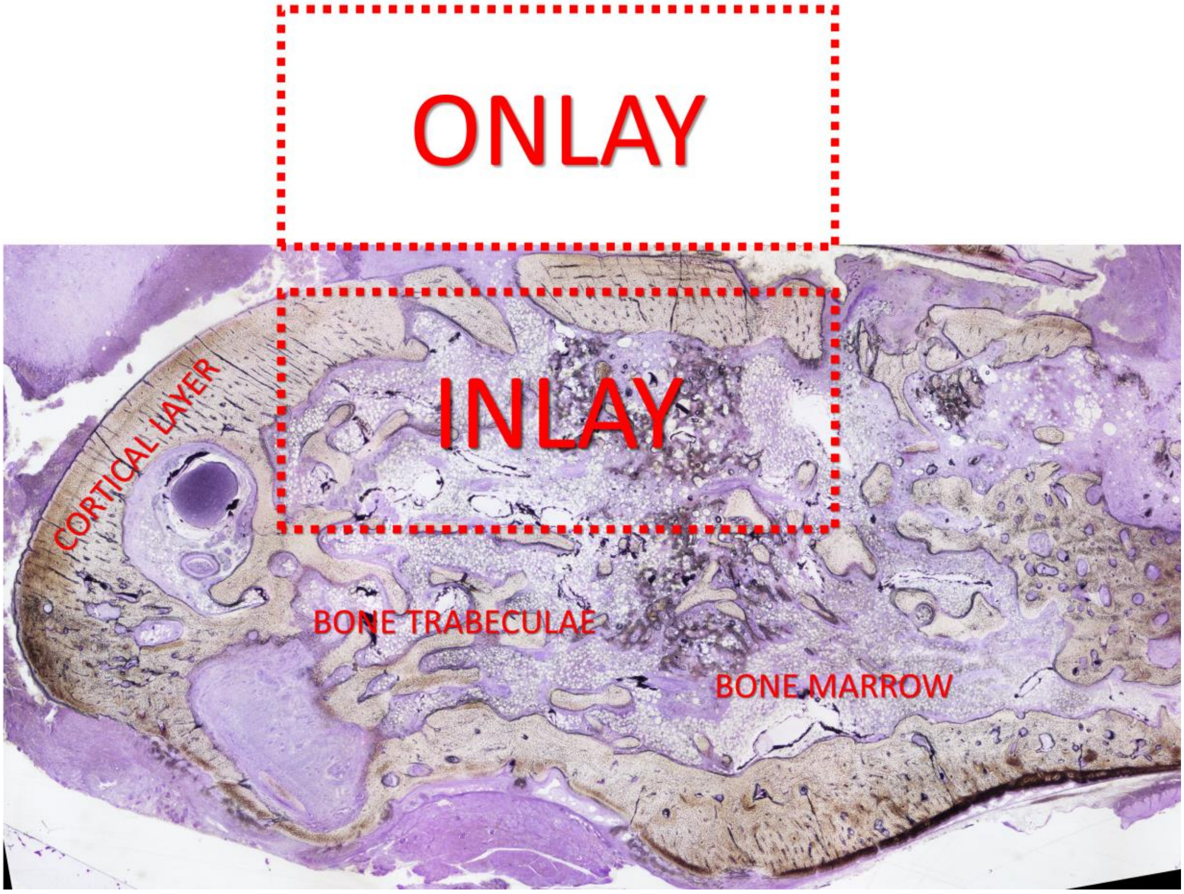

Figure S1. Photomicrograph of a ground section illustrating a section of a rabbit mandible with the various bone compartments. The dotted regions indicate the position of onlay/inlay grafts.
